# Rapid Assessment Zika Virus Knowledge Among Clinical Specialists in Singapore: A Cross-sectional Survey

**DOI:** 10.1371/currents.outbreaks.44b19196298e01f3a6dcd4c09f235fa8

**Published:** 2017-05-03

**Authors:** Chee F Yung, Clarence C Tam, Victor S Rajadurai, Jerry KY Chan, Mabel SF Low, Yong H Ng, Koh C Thoon, Lay K Tan

**Affiliations:** Infectious Disease Service, Department of Pediatrics, KK Women's and Children's Hospital, Singapore, Singapore; Saw Swee Hock School of Public Health, National University of Singapore, Singapore, Singapore; Department of Neonatology, KK Women's and Children's Hospital, Singapore, Singapore; Department of Reproductive Medicine, KK Women's and Children's Hospital, Singapore, Singapore; Saw Swee Hock School of Public Health, National University of Singapore, Singapore, Singapore; Department of Pediatrics, KK Women's and Children's Hospital, Singapore, Singapore; nfectious Disease Service, Department of Pediatrics, KK Women's and Children's Hospital, Singapore, Singapore; Department of Obstetrics and Gynecology, Singapore General Hospital, Singapore, Singapore

## Abstract

**Introduction:**

We report the results of a rapid assessment of Zika virus awareness among key clinical specialties in Singapore.

**Methods:**

Between June 6 and June 19, 2016 we conducted an online survey of doctors working in obstetrics and gynaecology, neonatology and paediatrics in Singapore. The survey included 15 multiple choice questions to measure respondents' knowledge of Zika virus in four domains covering clinical and public health.

**Results:**

A total of 110 survey responses (15% response rate) were obtained, 82% of respondents worked in the public sector. Overall, the median respondent score was 9.4 (Max score=15), with substantial variation (range: 3.5 - 14.7). Microcephaly and Guillain-Barré syndrome were recognised as causal complications of Zika virus infection by 99% and 50% of respondents respectively.  Clinical features which could help differentiate Zika from Dengue were less well understood with 50% and 68% correctly identifying conjunctivitis and low grade fever respectively. Worryingly, 14% favoured non-steroidal anti-inflammatory drugs as part of treatment, without first excluding dengue as a diagnosis. Also, only 36% of respondents were aware of the current recommendation for preventing sexual transmission of Zika virus. Fewer than 50% were aware of the need for ophthalmological evaluation as part of congenital Zika virus infection.

**Discussion:**

Our assessment demonstrates that there is good awareness of the clinical manifestation of Zika virus disease among key specialty doctors, but confusion with Dengue disease remains. It also highlights knowledge gaps in the prevention of sexually-transmitted Zika virus infection and the clinical management of congenital Zika virus infection in newborns. Our study identified strategic areas to improve communication to front-line doctors during public health response to the Zika epidemic.

## Introduction

On February 1, 2016, the World Health Organization (WHO) declared a Public Health Emergency of International Concern (PHEIC) in relation to the then suspected association between Zika virus infection and microcephaly.^1^ A causal association between Zika virus infection and microcephaly, and other neurological complications such as Guillain-Barré syndrome (GBS), has since been established on the basis of accumulating clinical and epidemiological evidence.^2^^,^^3^^,^^4^^,^^5^^,^^6^ Although the epidemic of microcephaly has been primarily focused in the northeastern part of Brazil, widespread international travel, the expansive geographic range of the Zika virus vectors, *Aedes aegypti* and *Aedes albopictus*, and increasing evidence of the potential for Zika virus transmission through sexual contact, means that spread of Zika virus and associated complications to new regions is of global concern. To date, 70 countries report ongoing mosquito-borne transmission of Zika virus, while microcephaly and other central nervous system malformations putatively associated with Zika virus infection have been reported from 20 countries.^7^ In four of these, microcephaly has been reported in foetuses or infants of mothers with a recent history of travel to a Zika-affected country.

Singapore, a tropical country with extensive international travel links, established *Aedes* mosquito populations and year-round, endemic transmission of dengue and chikungunya viruses, is at potentially high-risk for introduction of Zika virus. The first imported case of Zika virus was reported in May 13, 2016 in a 48 year-old man who had recently returned from Sao Paulo, Brazil. Subsequently, a local autochthonous Zika virus outbreak was detected in July/August 2016.^8^ However, genetic analysis suggests that the Singapore Zika virus outbreak strain was from Asia and did not originate from Brazil.^9^ Imported cases have also been reported in neighbouring countries with the potential to support autochthonous Zika virus transmission, and previous circulation of Zika virus has been documented in other nearby countries, including Cambodia, Thailand and Lao People's Democratic Republic.^10^

Since the PHEIC declaration, guidelines for the testing and clinical management of Zika virus disease and associated complications have been issued and repeatedly updated by WHO and the US Centers for Disease Control (CDC). In Singapore, the Ministry of Health (MOH) has sent out three information updates via email as well as letters to all registered doctors as of June 1, 2016, which included public health information such as mandating the notification of suspected Zika virus disease and interim clinical management guidelines for Zika virus disease in pregnant women.

Mass media and information access in Singapore are easily and conveniently accessible. As a dengue-endemic country, the risk of a Zika epidemic occurring has been reported by local media and the government. Medical practitioners are familiar with accessing information via the internet, including US CDC and WHO websites. Therefore, levels of Zika virus awareness among medical practitioners in Singapore should be representative of a standard that is realistically achievable in high-income countries as well as lower-resource settings with similar infrastructure for disseminating and receiving health information.

We report the results of a rapid assessment of Zika virus awareness among key clinical specialties in Singapore, with the aim of identifying gaps in knowledge and opportunities for improving communication on Zika virus disease management and control policies.

## Methods

**Study Participants and Sites**

Between June 6 and June 19, 2016 prior to the Zika virus outbreak, we conducted an online survey of doctors working in obstetrics and gynaecology, neonatology and paediatrics in Singapore. A link to the online survey was disseminated by email to the relevant doctors at KK Women’s and Children’s Hospital and Singapore General Hospital, two large public hospitals in Singapore. Together they cater for approximately 50% of women’s and children’s hospital care in Singapore. To capture responses from doctors working in the private sector, the survey was also sent to members of the Obstetrics and Gynaecology Society of Singapore and the College of Paediatrics and Child Health, Singapore. The survey was anonymous and could be completed in about 10 minutes on a computer or mobile device. In order to make the survey educational, respondents were given a summary of relevant recommendations after they responded to each question. Respondents did not receive any incentive to complete the survey.

Approval for this study, including waiver of informed consent, was obtained from the SingHealth Centralised Institutional Review Board (application number 2016/2251). The survey was anonymous so only anonymised data were used for analysis.

**Data Collection**

The survey included 5 questions regarding respondents' age group, sex, primary specialty, professional designation (Registrar/Resident, Associate Consultant or Consultant/Senior Consultant) and practice setting (public or private sector). In addition, 15 multiple choice questions were included to measure respondents' knowledge of Zika virus in four domains: (1) Clinical Presentation, Diagnosis and Management; (2) Transmission and Prevention; (3) Clinical Management of Pregnant Mothers Potentially Infected with Zika and Infants with Microcephaly; and (4) Ministry of Health Requirements for Notification of Suspected Zika Virus Infection. Of necessity, the survey was not an exhaustive set of questions, but was designed to capture general awareness of key knowledge and guidelines under these four domains. Respondents had to provide a response to every question in order to complete the survey.

**Data Analysis**

We summarised correct responses to each question using frequencies and percentages. In addition, survey responses were given a score of 0 to 15 based on the number of questions that were answered correctly. For some questions, respondents could choose more than one option (e.g. "Which types of mosquitoes are known to transmit Zika virus?"), so each question contributed a maximum of 1 mark to the total score, weighted by the number of possible correct options for that question. Scores were computed overall and for each of the four questionnaire domains. Statistical analysis was performed using Stata, version 11 (Stata Corporation).

To compare levels of knowledge across domains, we normalised scores for each domain by dividing each respondent's score by the maximum possible score for that domain, such that scores for each domain were scaled betwen 0 and 1. We summarised score distributions using the median, range and interquartile range, and compared scores between categories of respondent age group, sex, medical specialty, professional designation and public/private practice using boxplots and the Kruskal-Wallis test. Response rates were estimated using the number of email addresses the survey link was sent to as the denominator.

## Results

A total of 110 survey responses were obtained from emailing the survey to 747 doctors working in the key specialties of obstetrics and gynaecology, neonatology and paediatrics in Singapore. This represented an overall response rate of about 15%. Respondent characteristics are presented in Table 1. Approximately two-thirds of respondents were female, which is consistent with the gender distribution of doctors in the participating specialties, and 56% were aged 30 to 49 years; Residents and Registrars made up half the sample and 82% of respondents worked in the public sector. Neonatology was the most common specialty among respondents (56%).

**Figure:**
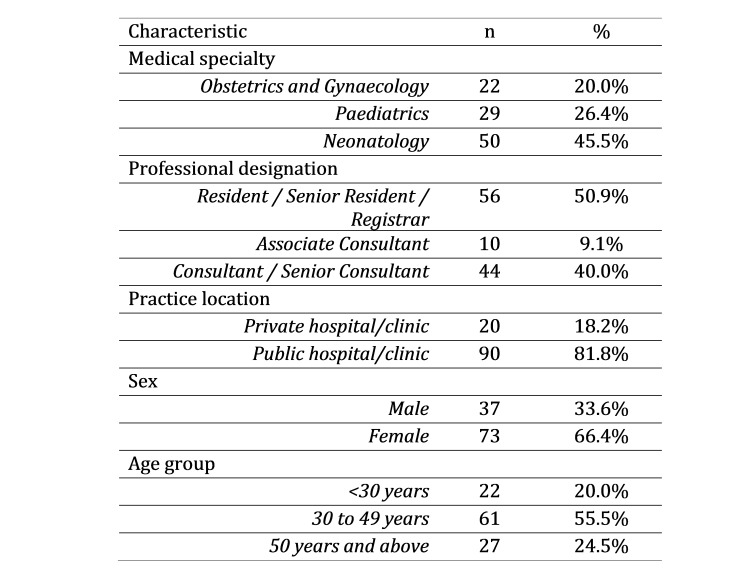
**Table 1:** Characteristics of doctors responding to a survey on Zika virus awareness, Singapore 2016 (n=110)

**Clinical Presentation, Diagnosis and Management**

Low-grade fever, rash and muscle or joint pain were each identified as typical symptoms of Zika virus disease by over 68% of respondents; fewer than 50% recognised conjunctivitis as part of the typical clinical presentation. Although signs of haemorrhage/bleeding and high-grade fever are not classical presentations of Zika virus disease, 6% and 37% of respondents respectively selected these. Microcephaly and GBS were recognised as a causal complications of Zika virus infection by 99% and 50% of respondents respectively.

The majority of respondents recognised blood (83%) and urine (48%) as primary and secondary samples for Zika virus testing. However, while 67% were aware that polymerase chain reaction (PCR) should be the first-line test for Zika virus identification within the first week of fever onset, 20% stated that both PCR and serology would be expected to be positive. In addition, 29% believed PCR and 40% believed both PCR and serology would also be expected to be positive beyond one week.

Nearly all respondents mentioned fever relief medication and fluid rehydration for management of Zika virus disease, but 14% favoured non-steroidal anti-inflammatory drugs, which are generally not recommended without first excluding dengue as a diagnosis.

**Figure:**
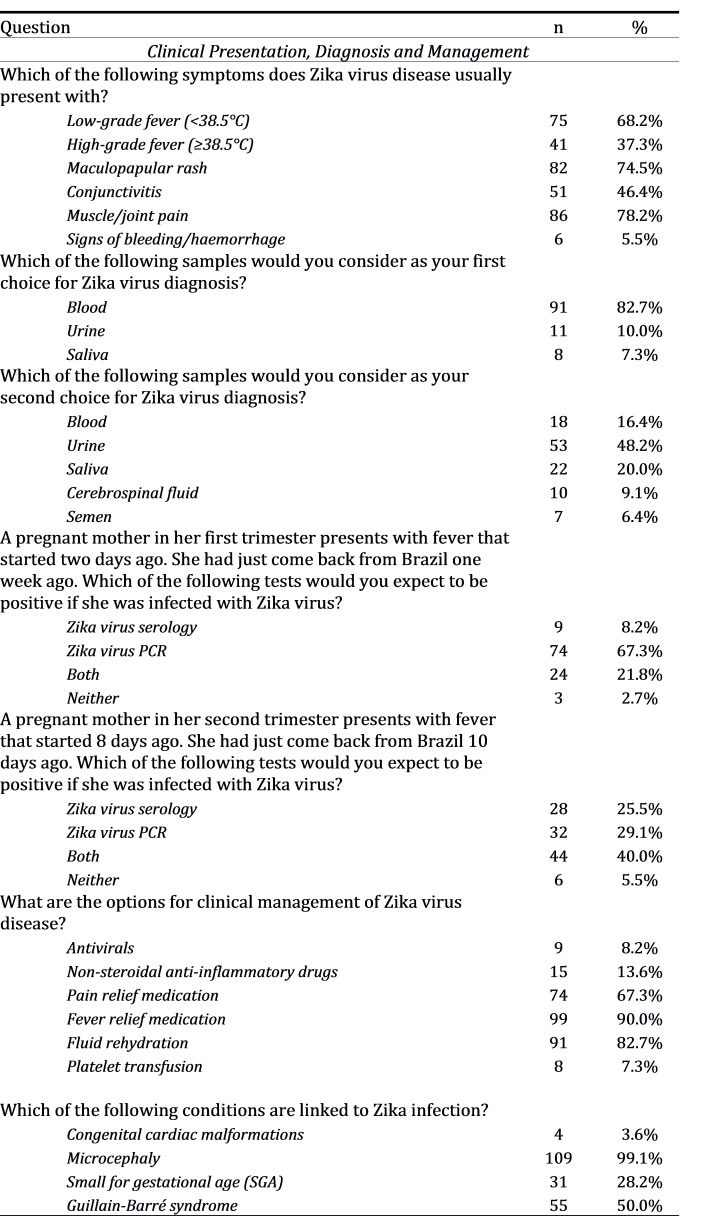
**Table 2A:** Responses to questions in a survey on Zika virus awareness among doctors (obstetrics and gynaecology, neonatology and paediatrics), Singapore 2016 (n = 110).

**Clinical Management of Pregnant Mothers Potentially Infected with Zika and Infants with Microcephaly**

82% correctly identified CDC's recommendation to test infants (or cord serum) for Zika virus, but only 16% recognised the need to additionally test for dengue virus. 42% believed specific testing of infant cerebrospinal fluid (CSF) for Zika virus was warranted; CDC guidelines indicate that CSF should be tested only if collected for other purposes.^11^

In addition, while 94% of respondents knew that cranial ultrasound was indicated for infants with suspected congenital Zika virus infection, only 57% recognised the need to test for other infections such as rubella, syphilis, toxoplasmosis and cytomegalovirus. Fewer than 50% were aware of the recommendation for ophthalmological evaluation within 1 month after birth.

**Figure:**
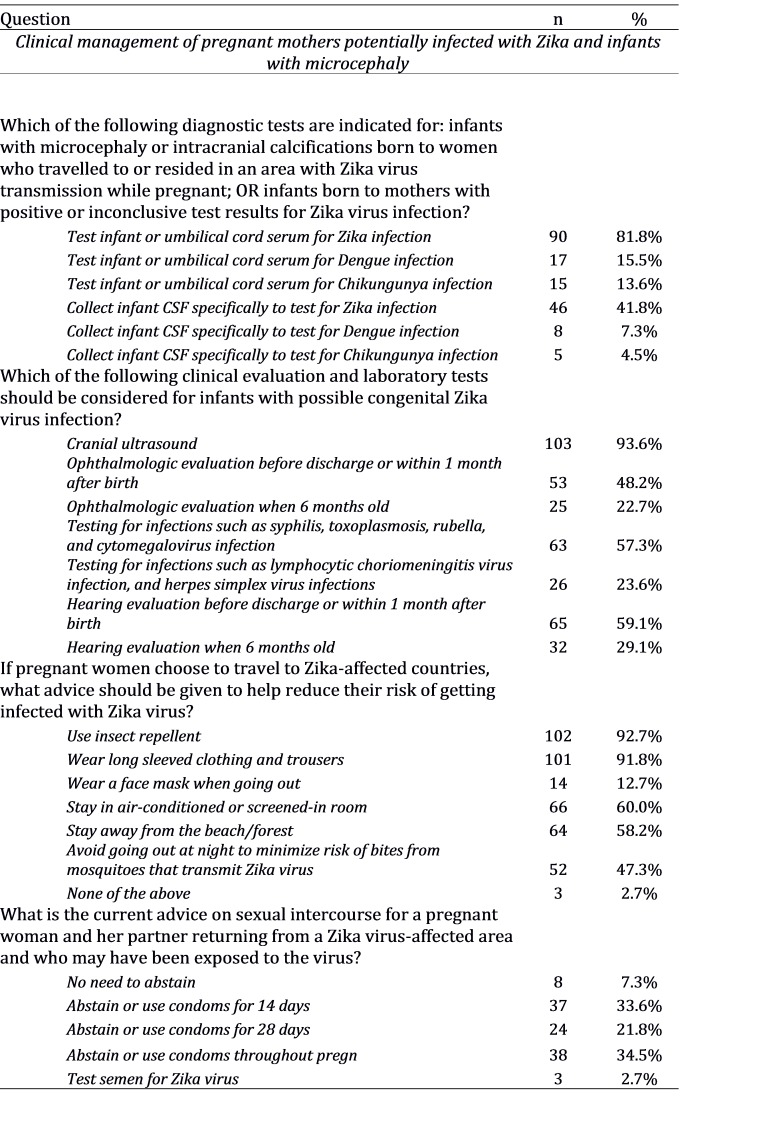
**Table 2B:** Responses to questions in a survey on Zika virus awareness among doctors (obstetrics and gynaecology, neonatology and paediatrics), Singapore 2016 (n = 110).

**Transmission and Prevention**

Most respondents (85%) identified* Aedes aegypti* as a mosquito vector for Zika virus, but only 32% knew that *Aedes albopictus* can also transmit the virus. Sexual contact and blood transfusion were recognised as alternative routes of transmission by 75% and 48% of respondents respectively, but 19% and 16% incorrectly mentioned droplet spread and saliva as transmission modes, and 34% mentioned breastfeeding.

Nearly all respondents were aware of personal measures to avoid *Aedes* mosquito bites among pregnant women travellers, such as use of insect repellent and wearing long-sleeved clothing. However, there were misconceptions regarding the location and timing of *Aedes* biting activity; 58% would incorrectly recommend staying away from beaches or forested areas and 47% would incorrectly recommend travellers to avoid going outdoors at night.

Only 36% of respondents were aware of the current recommendation for pregnant women and their partners returning from Zika-affected areas to abstain from sexual intercourse or use condoms appropriately throughout the duration of the pregnancy.

**Ministry of Health Requirements for Notification of Suspected Zika Virus Infection**

Among respondents, 81% were aware of the need to notify suspected cases of Zika virus disease even in the absence of microbiological confirmation, but there was lack of clarity regarding the definition of a suspected case of Zika virus disease; only 22% of respondents were able to correctly identify local MOH's definition of possible exposure to Zika virus.

**Figure:**
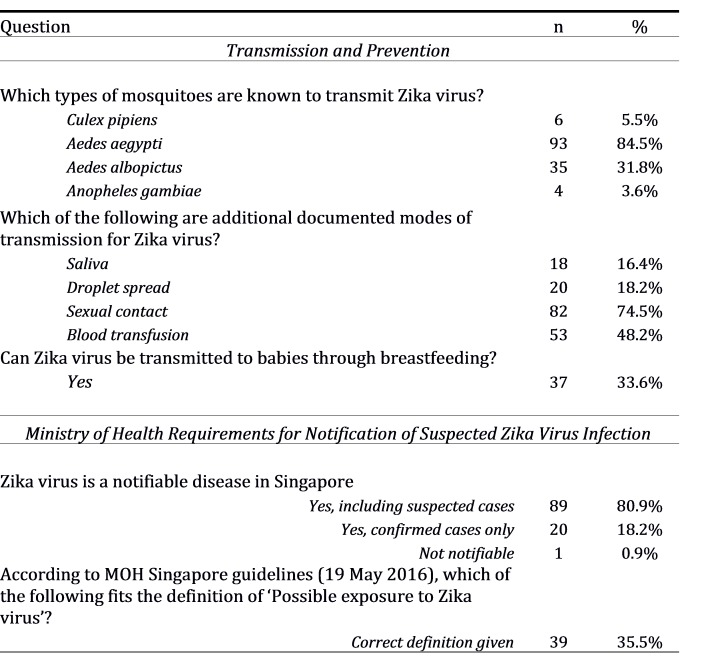
**Table 2C:** Responses to questions in a survey on Zika virus awareness among doctors (obstetrics and gynaecology, neonatology and paediatrics), Singapore 2016 (n = 110).

**Domain-level Knowledge**

Overall, the median respondent score was 9.4, with substantial variation (range: 3.5 - 14.7). Compared with the Clinical Presentation, Diagnosis and Management domain, median normalised scores were lower for domains on Transmission and Prevention (p<0.001) and Clinical Management of Pregnant Mothers Potentially Infected with Zika and Infants with Microcephaly (p<0.001) (Figure 1). There was no evidence of difference in scores by medical specialty, professional designation, respondent sex or age group, although statistical power for subgroup comparisons was low.

**Domain-level scores among respondents to survey of Zika virus awareness, Singapore 2016 (n = 110).**
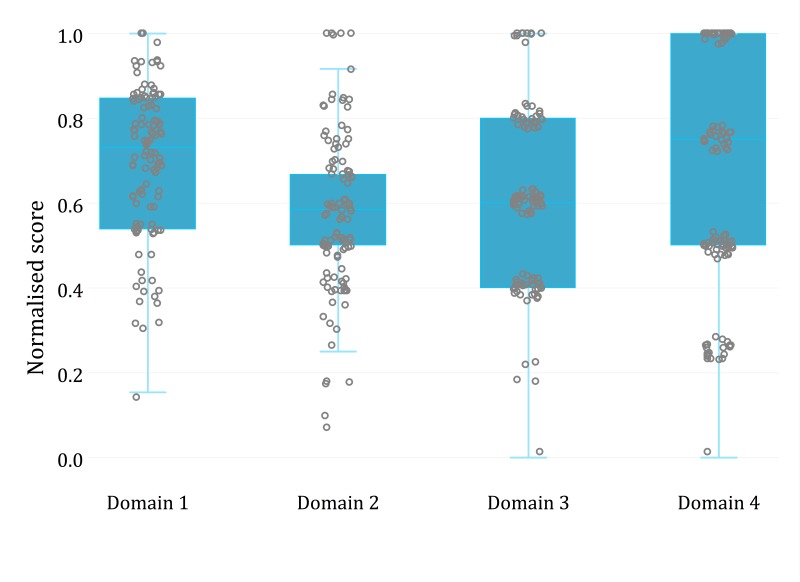
Domain 1: Clinical Presentation, Diagnosis and Management; Domain 2: Transmission and Prevention; Domain 3: Clinical Management of Pregnant Mothers Potentially Infected with Zika and Infants with Microcephaly; Domain 4: Ministry of Health Requirements for Notification of Suspected Zika Virus Infection

## Discussion

To our knowledge, this was the first such rapid assessment of key clinical specialty awareness of Zika virus disease guidelines at a national level globally. Our assessment demonstrated that there was good awareness of Zika virus disease signs and symptoms among doctors, but highlighted gaps in awareness of certain recommendations, particularly ones referring to the prevention of sexually-transmitted Zika virus infection in pregnant women, and the management of infants with possible congenital Zika virus disease. Our study identified opportunities for targeted improvement of communication around Zika virus guidelines among front-line doctors.

There was a high level of awareness among our sample of doctors with regards to three of the four common signs and symptoms of Zika virus disease. These three signs and symptoms are similar to those for dengue, which is endemic in Singapore. It was likely that the numerous public health and educational campaigns on dengue may have aided the translation of clinical dengue knowledge to Zika virus disease. This could also explain the lack of awareness that conjunctivitis, which is less common in dengue cases, is an important distinguishing clinical feature of Zika virus disease. Similarly, 35% of respondents incorrectly chose ‘high-grade fever’ as a feature of Zika virus disease. These findings suggest that while knowledge of signs and symptoms of Zika virus disease that are similar to dengue may be good, there remains a gap in understanding of clinical features that could help differentiate Zika virus disease from dengue. This was not surprising since most information outlets have repeatedly reported the similarities between the Zika and dengue viruses, which belong to the same flaviviridae virus family and share common mosquito vectors. Furthermore, messages issued by most public health bodies to clinicians provide standard case definitions that do not highlight the distinguishing features of Zika virus disease. There may be a need to change the way such messages are conveyed, with more emphasis given to differentiation rather than a standard listing of signs and symptoms.

We detected similar confusion in the clinical management of Zika virus disease that could be attributed to its perceived similarity to dengue. While >85% of respondents correctly stated fever relief medication and fluid rehydration as the main treatment options, a small minority (7%) wrongly chose platelet transfusion as a possible treatment for Zika virus disease. Worryingly, 13% stated that they would consider NSAIDS for managing Zika virus disease. This is not routinely recommended, as consumption of NSAIDS could have grave consequences if a patient with dengue was misdiagnosed as having Zika virus disease. When stratified by medical specialty, the highest percentage selecting this option worked in Obstetrics and Gynaecology (23%). We were not able to exclude the possibility that the respondents who selected this option were aware of the need to exclude dengue before prescribing NSAIDs.

Two-thirds of respondents knew that PCR would likely be positive during the first few days of disease onset, but one-fifth wrongly stated that both PCR and serology testing would be positive. In a follow-up question with a more complicated scenario involving disease onset more than 8 days ago, 40% stated that both PCR and serology would be expected to be positive, while 25% wrongly selected PCR as the test of choice. Taken together, these findings suggest there is a need to improve the level of understanding for Zika virus disease diagnostics. Understanding the utility of PCR and serology in relation to the onset of symptoms is critical to minimise the potential for false positive and false negative diagnoses.^12^ This technical understanding is needed when developing follow-up management plans for patients. It is also fundamental in helping to effectively communicate the uncertainties of laboratory results to concerned patients.

Nearly all respondents correctly identified microcephaly as a condition linked to Zika virus disease. However, only 50% were aware of the confirmed link with GBS.^5^^,^^6^^,^^13^ This may be due to the fact that our sample’s medical specialties were related to foetal, infant and paediatric patients, for which microcephaly is a key area of interest. However, 27% also stated incorrectly (at the time of the survey) that small-for-gestational age (SGA) was also linked to Zika virus disease. There is now increasing data supporting a link between Zika virus disease and SGA or Intrauterine growth retardation.^13^ Our survey was based on available information during June 2016, which pointed to a more targeted neurotropic effect preferentially affecting tissues of the brain and brain stem from Zika virus infection during pregnancy.^14^

Our analysis also points to gaps in knowledge regarding recommendations to prevent Zika virus infection in pregnant women. In particular, only one third of respondents were aware of the current guidelines recommending that pregnant women and their partners returning from Zika-affected areas practise safe sex or abstain from sexual intercourse for the duration of the pregnancy. It was reassuring to find that a high proportion of doctors (>90%) gave correct advice in terms of recommending the use of repellents and long-sleeved clothing to minimise the risk of mosquito bites. However, about half of respondents had misconceptions regarding biting behaviour of *Aedes* vectors, such as preference for night biting or beach/forest settings. This was surprising considering the numerous dengue education campaigns implemented by the authorities in Singapore, although the focus of these campaigns has been primarily on source eradication to prevent mosquito breeding.^15^

**Limitations**

We intentionally targeted the survey only at the clinical specialties of obstetrics and gynaecology, neonatology and paediatrics since the nature of the Zika epidemic was most relevant to their patient cohort. Furthermore, in Singapore, pregnant mothers are routinely managed and followed up by obstetricians rather than primary care physicians. Therefore, our results should be considered ‘best case’ scenarios in terms of knowledge levels amongst doctors.

We chose to conduct an online survey as this is a rapid and convenient mode of administration. KK Women’s and Children’s Hospital is the only public hospital catering specifically only for neonatal, paediatric, obstetrics and gynaecology care in Singapore. We believe that our email list reached a significant proportion of doctors practising in the relevant specialties by also including the Singapore General Hospital (the largest general public hospital in Singapore) as well as those in private practice via the medical societies and colleges. However, we cannot be sure that all the relevant doctors in Singapore were aware of the survey. Of note, participation from the private sector was low, with doctors in private practice comprising only 18% of respondents. We did not detect any obvious difference between public and private sector doctors but our study was not powered for such detailed analysis. However, we do not think awareness and knowledge levels would be lower amongst private practitioners as they also receive the same updates via email and SMS from the Ministry of Health.

We restricted the time period of the survey to two weeks, to minimise the possibility of official guidelines being altered during the survey period, as public health authorities are continuously reviewing and updating recommendations. During the survey period, the WHO updated its recommendations on sexual transmission of Zika virus, but these did not influence any of the questions in our survey.

Our sample size also precluded robust comparison of knowledge between clinical specialties and medical designations, although this was not a primary objective. Despite this, our survey has enabled rapid identification of gaps in knowledge that can be acted upon to improve local as well as global preparedness for management and control of Zika virus disease.

**Recommendations**

Our rapid assessment identified good levels of Zika virus disease knowledge among doctors in key clinical specialties of obstetrics and gynaecology, neonatology and paediatrics in Singapore. The findings provide a reference threshold in terms of knowledge level among doctors in settings with robust infrastructures to access information for a rapidly-evolving epidemic. Knowledge levels in lower-income countries with limited public health systems and access to information via the internet may be much lower. We have identified specific key areas for targeted improvements when communicating Zika virus disease knowledge to frontline doctors. We recommend that future guidelines emphasise clinical signs and symptoms which differentiate Zika virus disease from similar conditions such as dengue, particularly in countries with endemic dengue virus transmission. Similarly, greater emphasis in information sources on not using NSAIDS as a treatment option unless dengue has been excluded is also needed. There is an urgent requirement to improve doctors' knowledge on the need to advice at risk patients to practise safe sex or abstain from sexual intercourse for the duration of the pregnancy. Similarly, it is important to highlight the importance of ophthalmic evaluations in congenital Zika virus infection. In addition, there is still a need to educate doctors in key specialties regarding the appropriate use of Zika virus diagnostic tests and their current limitations, both to minimise the risk of misdiagnosis and to help patients make informed decisions.

## Competing Interests

The authors have declared that no competing interests exist.

## Corresponding Author

Clarence Tam: clarence.tam@nus.edu.sg

## Data Availability

Data are available through Figshare at https://figshare.com/s/2ff4859475c8914d8770
